# Bean‐based nutrient‐enriched puffed snacks: Formulation design, functional evaluation, and optimization

**DOI:** 10.1002/fsn3.1727

**Published:** 2020-08-08

**Authors:** Hedwig Natabirwa, Dorothy Nakimbugwe, Mercy Lung'aho, Kashub S. Tumwesigye, John H. Muyonga

**Affiliations:** ^1^ School of Food Technology Nutrition & Bioengineering Makerere University Kampala Uganda; ^2^ National Agricultural Research Laboratories National Agricultural Research Organization Kampala Uganda; ^3^ Centre for International Tropical Agriculture Kampala Uganda

**Keywords:** acceptability, Bean snack, extrusion, nutrient contribution, optimization, protein digestibility, texture

## Abstract

School‐age children frequently consume snacks. However, most of the snacks they consume are of low nutritional quality. The objective of this study was to develop a nutrient‐rich and acceptable extruded bean‐based snack, which could contribute to improved nutrient intake, especially for school‐age children. Snack formulations developed from Roba1 beans, maize, orange‐fleshed sweet potato, and amaranth mixtures, and processed in a twin‐screw extruder, were evaluated and optimized for nutritional, textural and sensory properties. High proportion of beans in the formulation was associated with high protein, iron, zinc, and dietary fiber content. An optimal formulation (82.03:10: 5:2.97; beans, maize, OFSP, amaranth), containing 20.38 g, 4.12 g, 4.83 mg, and 1.51 mg per 100 g, of protein, dietary fiber, iron, and zinc, respectively, was obtained. The snacks were crunchy and moderately acceptable with average sensory scores of 6 on a 9‐point hedonic scale, and hardness 26.6 N. Nutrient contribution 43, 19, and 12% for protein, iron, and zinc, respectively, to children aged 6 to 8 years; and 24, 19, and 7.6%, respectively, to children aged 9 to 12 years from a 40 g serving was estimated from the snack. The results demonstrate the potential of using extrusion to produce nutrient enriched value‐added food products from blends of iron‐rich beans and common staples.

## Graphical abstract

Nutrient‐rich extruded composite snack formulations were processed from common beans, maize, orange‐fleshed sweet potato, and amaranth and evaluated. An optimal composite snack with acceptable taste, texture, and high potential nutrient contribution in terms of protein, iron, and zinc (to children) was identified.

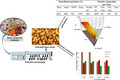

## 1 BACKROUND

Nutrient‐enriched foods complement both nutritional and developmental needs of children (Bhutta et al., 2013; Dewey, 2013). Such nutrient‐enriched foods can easily be delivered by enhancing popular conventional snacks with abundant and unexplored nutrient‐rich foods. Snack foods remain popular with consumers especially children and travelers and are rich sources of energy. Since snacks are consumed regularly, they hence have great potential to deliver nutrients. Globally, snack consumption has significantly increased over the last few decades and is projected to continue rising (Choi, Phillips, & Resurrection, 2007; Patel, Patel, & Singh, 2016; Shriver et al., 2017). In the United States for instance, the contribution of snack foods to daily energy intake among children aged just 2–5 years was estimated as 28% (Shriver et al., 2017).

Although snacks are filling and help to meet the daily energy needs, they generally lack a variety of nutrients, such as iron, zinc, protein, and vitamin A, which are essential for active growing children (Korkerd, Wanlapa, Puttanlek, Uttapap, & Rungsardthong, 2016). Consequently, snack dependence, especially during school time, has been one of the key drivers for increasing nutrient deficiency among children (Korkerd et al., 2016). However, the nutritional quality of snack foods can be improved through blending different foodstuffs and fortification. Widely available nutrient‐rich foods such as common beans could be used to enrich snacks. Studies (Nyombaire, Siddiq, & Dolan, 2011; Siddiq, Kelkar, Harte, Dolan, & Nyombaire, 2013) reported significant improvements in nutrient content when common beans were blended with cereal foods.

Processing technology is another important consideration in the manufacture of snacks. Industrial production of ready‐to‐eat snacks using extrusion technology has been reported to yield shelf‐stable, hygienic, and acceptable foods (Reddy et al., 2014). Previous studies have demonstrated the potential of extrusion to produce expanded snacks and instant flours from legumes (Nyombaire et al., 2011; Siddiq et al., 2013; Simons et al., 2015). Acceptable extruded food puffs were developed from navy and pinto beans by Simons et al. (2015). However, very limited work exists on extruded snacks from iron‐rich beans. New iron‐rich bean composite products especially using controlled and optimized extrusion processes may enhance product diversity, commercialization, consumption, and nutrient intake. The study therefore was aimed at developing a protocol for production of an extruded protein‐, iron‐, and zinc‐rich snack from Roba1 beans and other ingredients, and evaluating its potential contribution to nutrient intake among school‐age children aged 6–12 years.

## 2 MATERIALS AND METHODS

### 2.1 Raw material preparation

Newly harvested Roba1 beans, an iron‐rich landrace bean variety, were obtained from Community Enterprise Development Organization (CEDO), Rakai district, Uganda. The beans were included as the main source of protein, iron, and zinc. Beans also contain high levels of lysine compared with maize (USDA, 2019). Dry amaranth grain, maize grain, and fresh orange‐fleshed sweet potato (OFSP) were obtained from farmers in Mukono district, Uganda. OFSP was included as a source of pro‐vitamin A beta carotene (Bengtsson, Namutebi, Alminger, & Svanberg, 2008), as well as to enrich the color and appearance of extrudate. Amaranth is still an underutilized food crop, but also rich in iron and zinc (Muyonga, Andabati, & Ssepuuya, 2014; USDA, 2018). Maize, amaranth and sweet potato were included as sources of starch (a key ingredient in puffed snacks), and to enhance product palatability.

Roba1 beans were sorted to remove any foreign matter (stones, chaff, and plant parts), washed with clean potable water, and dried in a hot air oven (50–55°C, 16 hr) to 8%–10% moisture content. OFSP was washed with clean potable water, peeled, thinly sliced (approx. 5 mm width × 2 mm, thickness chips), and oven‐dried (50–55°C, 8 hr). The beans, maize, amaranth, and OFSP were separately milled into flour using a commercial mill (Model YZMF, Yize, Shuliy Henan, China), to pass through a 1.5 mm sieve.

### 2.2 Snack formulation

Bean‐based snacks were prepared from beans, maize, OFSP, and amaranth flour. In total, 20 formulations were generated using mixture design using Design–Expert software (version 11, Stat‐Ease Inc.), five of which were replicates. A formulation that could provide the highest protein, iron, zinc, and dietary fiber content and suitably contribute to the recommended daily intakes for children aged 6–12 years (Whitney & Rolfes, 2011) was determined by numeric optimization using the Design–Expert software.

### 2.3 Extruded snack processing

Snack samples were processed using a Twin Screw extruder (Model DP 70‐III) at barrel temperatures 60/120/142°C for the feed, internal barrel and die temperatures, respectively, and feed moisture 15% as determined in previous works (Natabirwa, Nakimbugwe, Lung’aho, & Muyonga, 2018). The screw speed, feed rate, and cutter speed were 45 Hz, 30 kg/hr, and 18 Hz, respectively. The extrusion feed material for making expanded extruded snacks was preconditioned with water to achieve a uniform moisture content of 15% (Natabirwa, Muyonga, Nakimbugwe, & Lungaho, 2018; Natabirwa, Nakimbugwe, et al., 2018; Steel et al., 2012). The extruder die diameter, flighted length of screw, screw diameter (*d*), outer screw diameter (*D*), and length to diameter of extruder were 6 mm, 124 mm, 27 mm, 41 mm, and 18:1, respectively. Cylindrical shaped extrudates (3 cm length and relative radial expansion ratio of 4) were obtained.

The developed snack extrudates were dried in a Multi‐Layer VTO dry air oven (DP‐DKX‐II, 85°C, 12 min.) to final moisture content of 6%, and flavored (with chicken flavor, Afribond, South Africa). The snacks were double‐layer packaged in high‐density polythene (gauge 30 microns) to prevent moisture and air entry, and stored (at 25–28°C) until further analysis.

### 2.4 Extruded snack analysis

#### 2.4.1 Determination of nutrient content

Nutrient composition data for the different formulations were computed using a Nutrisurvey programming tool (Erhardt, 2007), based on nutrient composition data in food databases (USDA, 2018) and that previously obtained for Roba1 beans (Natabirwa, Muyonga, et al., 2018; Natabirwa, Nakimbugwe, et al., 2018). The crude protein, ash, dietary fiber for optimized extrudate formulation were, respectively, determined using the Kjeldahl method (AOAC, 2005), dry ashing at 550°C and acid detergent fiber methods (Shimelis & Rakshit, 2005). Protein content was calculated using Equation 1.

(1) Protein content=Total nitrogen×6.25

#### 2.4.2 Protein digestibility

Protein digestibility was determined using the multi‐enzyme (trypsin‐chymotrypsin‐papain) technique (Hsu, Vavak, Satterlee, & Miller, 1977). The multi‐enzyme technique involved use of porcine pancreatic trypsin (Type IX) with 13,400 units/mg of protein, bovine pancreatic chymotrypsin (Type II) with 96 units/mg of protein, and papain (1.5–10 units/mg). The pH decrease over a 10 min period was recorded with a pH meter. The percentage of protein digestibility (*Y*) was calculated using Equation 2.

(2) Y=210.46-18.10X,

where, *X* was the pH after 10 min.

#### 2.4.3 Texture analysis

The texture of cylindrical extrudates (average, 3 cm long pieces) was measured using a Stable Microsystems Texture Analyzer (Model TA.XT‐Plus 42095) by compression with a cylindrical probe of 6 mm diameter (SMS P/6) following methods used earlier (Natabirwa, Nakimbugwe, et al., 2018). Hardness was taken as the maximum force (*N*) required to break the extruded samples, while crunchiness/crispiness was estimated from the energy required to puncture the extrudate (*N*.mm), equivalent to the average area under the force deformation curve (Ding, Ainsworth, Plunkett, Tucker, & Marson, 2006). The test speed was 2 mm/s and the penetration distance was 5 mm, with a trigger force of 0.049 N. The return distance of the probe was kept at 20 mm. A force‐time curve was recorded and analyzed by Texture Exponent 32 software program. Ten measurements were performed on each sample and averaged.

#### 2.4.4 Sensory evaluation

Extruded bean‐based snack collets were subjected to acceptability evaluation in the laboratory using 52 consumer panelists (both male and female) aged 18–30 years. Laboratory panelists were students of food science and technology. Prior to evaluation, panelists signed consent forms for voluntary participation. A 9‐point hedonic judgement scale was used for score, where 1 was “*dislike extremely*” and 9 “*like extremely*” (Watts, Ylimaki, Jeffery, & Elias, 1989). Attributes tested were appearance, flavor, taste, hardness, crunchiness, and overall acceptability.

#### 2.4.5 Snack acceptability among school children

Two snack formulations with high scores overall from adults evaluation and also based on percentage of beans in the formulation (66% and 85%, respectively) were presented for consumer evaluation by school children. A panel of 112 children aged 8–12 years (who could easily read and understand the questionnaire) evaluated the snacks for taste and texture against a 5‐point pictorial hedonic scale, where 1 indicated “*dislike very much*” and 5 “*like very much*” (Guinard, 2001). Forms of consent were duly signed by the parents and teachers on behalf of the children prior to sensory evaluation.

### 2.5 Statistical analysis and formulation optimization

Mixture design with numeric optimization (Design Expert 11; Stat‐Ease Inc.) was used for experimental design and analysis (Zhou, Liu, Dong, & Jiang, 2007). Means of values were computed using Minitab 16. Data were modeled by multiple regression analysis for mixture designs using linear and quadratic models (Equation 3 and 4). The statistical significance of terms was examined by analysis of variance for each response (Balasubramanian, Kaur, & Singh, 2014; Myers, Montgomery, & Anderson‐Cook, 2016). It was assumed that n mathematical functions existed: $f_{k}\;\left(k\;=\;1,\;2,\;3,\;\ldots ,\;n\right)$.

Linear,

(3) $$Y_{k}\;=\;\sum_{i=1}^{4}B_{i}X_{i}.$$

Quadratic,

(4) $$Y_{k}\;=\;\sum_{i=1}^{4}B_{i}X_{i}\;+\;\sum_{1\leq i<j}^{4}B_{\mathrm{ij}}X_{i}X_{j},$$

where, *Y_k_* = response variable, *B_i_*, *B_ii_*, and *B_ij_* are linear, quadratic, and interaction regression coefficients. *X_i_* and *X_j_* are independent variables.

The goodness‐of‐fit of the models was evaluated using the significance of lack‐of‐fit, correlation coefficients (*R* ^2^), adjusted *R* ^2^ values, and derived model *p*‐values. The criterion for optimizing formulations was based on numerically maximizing, minimizing, or targeting parameters for each response variable (protein, fiber, iron, zinc, taste, flavor, and texture) depending on the perceived need for obtaining desirable snacks. Optimization of ingredient levels was done by selecting responses, on the basis that they had direct effect on the nutritional quality and acceptability of the snack formulation as shown by their respective regression (*R* ^2^) values. Optimal formulations for production of a snack with high protein, iron, dietary fiber, and zinc content as well as a soft crunchy texture were identified.

## 3 RESULTS AND DISCUSSION

### 3.1 Effect of ingredient composition on nutrient content

**FIGURE 1:**
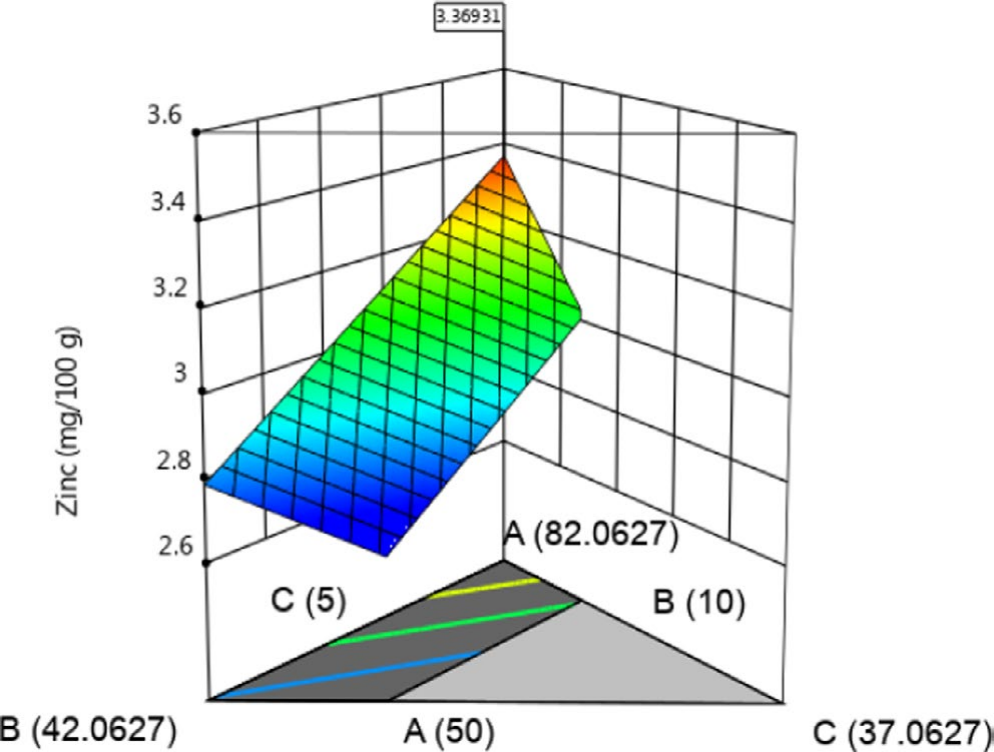
Response surface curve for the effects of ingredient proportions on bean‐based extruded snack zinc content and optimal value. A, beans; B, maize; and C, OFSP; where amaranth was kept constant at 2.94 g/100 g of total ingredients

The nutrient composition per 100 g for bean snack formulations is presented in Table 1. Increases in protein, iron, and zinc content were associated with high proportion of beans within a formulation (Table 1; Figure 1). Further, regression analysis (Table 2) showed that change in the amounts of beans, maize, amaranth, and OFSP had significant linear effect (*p* < .05) on the protein, dietary fiber, iron, and zinc content of the snack extrudates. Coefficients of determination (Table 2) for the linear and quadratic models (*R* ^2^ values) implied that 99% of the changes in protein, 92% for dietary fiber, 92% changes of iron, and 98% zinc content among the formulations were attributable to ingredients. High proportion of OFSP and maize content in the formulation resulted in increasing total carbohydrates content (Table 1), while fiber content was associated with beans and amaranth content. Common beans and amaranth are generally rich in protein, iron, and zinc content (USDA, 2018), which could explain the increases within formulations.

**TABLE 1:** The nutrient composition (estimated), protein digestibility and texture properties of the bean‐based snack formulations

Exp. run	Ingredient proportions				Nutrient composition per 100 g							Prot. dig. (%)	Instrumental Texture values	
	Beans (%)	Maize (%)	OFSP (%)	Amar (%)	Protein (g)	Energy (kcal)	CHO (g)	Diet‐fibre (g)	Fat (g)	Fe (mg)	Zinc (mg)		Hardness (*N*)	Area‐FD (*N*.mm)
1	50.00	34.33	10.66	5.01	16.60	388.80	66.90	11.30	2.60	4.70	2.70	71.50	46.40	177.00
2	50.00	34.33	10.66	5.01	15.50	353.10	68.50	8.50	2.80	4.10	2.70	74.68	45.45	197.04
3	50.00	34.33	10.66	5.01	17.00	353.00	67.50	8.60	3.20	4.50	2.90	74.38	51.25	157.17
4	50.00	45.00	5.00	0.00	16.70	390.80	72.10	11.70	2.80	4.60	2.80	75.10	58.40	137.70
5	52.14	22.86	15.00	10.00	17.00	385.40	62.50	11.10	2.50	5.00	2.70	73.20	45.30	171.70
6	54.04	30.56	5.40	10.00	18.00	383.20	68.40	11.80	2.90	5.30	2.90	72.80	46.20	197.60
7	55.66	29.34	15.00	0.00	17.20	387.90	63.80	11.60	2.20	4.60	2.70	74.70	53.00	242.90
8	59.25	35.75	5.00	0.00	18.30	385.10	69.50	12.40	2.50	5.00	2.90	78.20	52.80	201.90
9	60.66	14.34	15.00	10.00	18.60	379.70	59.80	11.80	2.30	5.30	2.90	72.90	45.20	139.30
10	60.94	24.06	5.00	10.00	19.20	378.70	66.40	12.30	2.70	5.50	3.00	73.30	37.40	154.10
11	65.55	17.70	15.00	1.75	18.90	381.20	60.70	12.20	1.90	5.10	2.90	73.20	43.20	181.50
12	65.99	25.17	8.84	0.00	19.30	381.00	64.80	12.60	2.10	5.20	3.00	74.40	51.30	157.20
13	65.99	25.17	8.83	0.00	17.40	347.80	66.40	10.50	2.30	4.30	3.03	74.19	53.83	223.43
14	68.02	22.64	5.00	4.34	20.20	377.30	65.80	12.90	2.30	5.50	3.10	73.70	48.50	171.00
15	68.02	22.64	5.00	4.34	18.20	347.80	65.40	10.90	2.40	4.70	3.11	73.17	45.34	171.72
16	71.85	10.00	8.61	9.54	20.90	372.60	60.80	12.90	2.20	5.80	3.20	74.26	43.80	182.40
17	71.85	10.00	8.61	9.54	18.80	346.60	65.10	12.00	2.30	4.90	3.14	72.87	45.15	139.33
18	72.35	10.00	13.95	3.70	20.30	375.60	58.80	12.70	1.80	5.50	3.00	72.50	45.10	167.80
19	76.44	16.78	5.83	0.95	21.40	373.80	63.60	13.50	2.00	5.70	3.20	72.50	54.60	201.10
20	85.00	10.00	5.00	0.00	23.00	368.50	61.90	14.20	1.70	6.00	3.40	74.30	43.00	139.30

Abbreviations: Area‐FD, area‐to‐force dimension ratio; CHO, carbohydrate; Fe, iron; Hd, hardness; Prot. dig., protein digestibility.

**TABLE 2:** Estimated regression coefficients and coefficients of determination for the effects of ingredients on extruded bean composite snack attributes

Factors	Protein	Dietary fiber	Iron	Zinc	Taste	Hd (*N*)	Area‐FD ratio
*X*_1_	25.99***	14.22***	6.06***	37.56***	7.31***	0.132	174.92
*X*_2_	6.70***	11.82***	4.54*	19.38*	19.86***	0.153	136.43
*X*_3_	24.10***	13.25***	3.75*	2.44*	13.31**	−54.48	−660.37
*X*_4_	72.36***	8.35***	6.19**	27.55**	−99.15*	53.22	40.92
*X*_1,2_	3.22	−1.33			−23.93	−0.01	83.94
*X*_1,3_	−25.93	−5.48			−3.37	0.67**	1,240.74**
*X*_1,4_	−63.27	4.50			136.47	−0.84*	87.94**
*X*_2,3_	−28.44	4.09			−34.49	0.85***	1,379.27*
*X*_2,4_	−66.51	3.96			109.24	−1.72	335.42
*X*_3,4_	−100.69	−1.27			−75.55	−1.40	559.90
*R* ^2^	0.99	0.92	0.92	0.98	0.61	0.93	0.65
*R* ^2^ adjusted	0.99	0.91	0.90	0.98	0.25	0.78	0.34
Model lack‐of fit *p*‐values	0.98	0.96	0.98	0.50	0.79	0.45	0.85

Note

Regression coefficients are significant, *p ≤ .05; **p ≤ .01; ***p ≤ .001.

Abbreviations: area‐FD, area‐force dimension ratio; Hd, hardness; *X*_1_, beans; *X*_2,_ maize; *X*_3_ OFSP; *X*_4_, amaranth.

### 3.2 Protein digestibility

Extruded formulations containing beans, maize, OFSP, and amaranth produced snacks with higher protein digestibility (PD; ≥71.5%; Table 1), as compared to raw ingredients whose PD ranged from 50.6% to 62.4%. The high PD could be due to denaturation of protein during extrusion heating (Alonso, Aguirre, & Marzo, 2000; Martín‐Cabrejas et al., 2009). Formulations containing relatively high maize content generally exhibited high PD, which might be associated with the protein‐starch interactions that increase the hydrophilic groups and hence the molecular solubility of protein (Siddiq et al., 2013). However, decrease in protein digestibility observed among formulations with high protein content could be due to conformational changes in the protein structure and protein re‐aggregation (Krupa‐Kozak & Soral‐Śmietana, 2010). Changes in protein structure causing relatively low PD at high extrusion temperature may suggest the need for extrusion of bean formulations at low temperatures as recommended by Anton, Fulcher, and Arntfield (2009) and Siddiq et al. (2013). Siddiq et al. (2013) reported that high extrusion temperatures reduce PD of foods. Relatively low PD in general might also be due to presence of phytates, polyphenols, tannins, and protease inhibitors, which interact with proteins forming complexes (Konietzny, Jany, & Greiner, 2006). Regression coefficients showed that variation in ingredient proportions had no significant influence (*p* > .05) on the protein digestibility of extrudates. Thus changes within the physicochemical components and other factors in extrusion may explain the changes in extrudate protein digestibility.

### 3.3 Extruded bean‐based composite snack extrudates texture

Considerable variations in hardness and crunchiness (measured as area‐force dimension ratio) of the composite bean snack extrudates (Table 1) were observed, which seemed dependent on raw material composition in a formulation. The high protein content was associated with crunchy extrudates, which could be due to the protein‐starch interactions that lead to formation of numerous tiny air cells in the extrudate (Onwulata, Smith, Konstance, & Holsinger, 2001). Results agree with previous work (Onwulata et al., 2001) which explained that increases in protein content in extrudates, may increase the crunchiness and crispiness of extrudate. Thus, high protein‐high starch content within bean ingredients is likely to improve the texture of snack extrudates.

### 3.4 Extruded bean‐based composite snacks acceptability

#### 3.4.1 Extruded bean‐based snack acceptability by adult panelists

**FIGURE 2:**
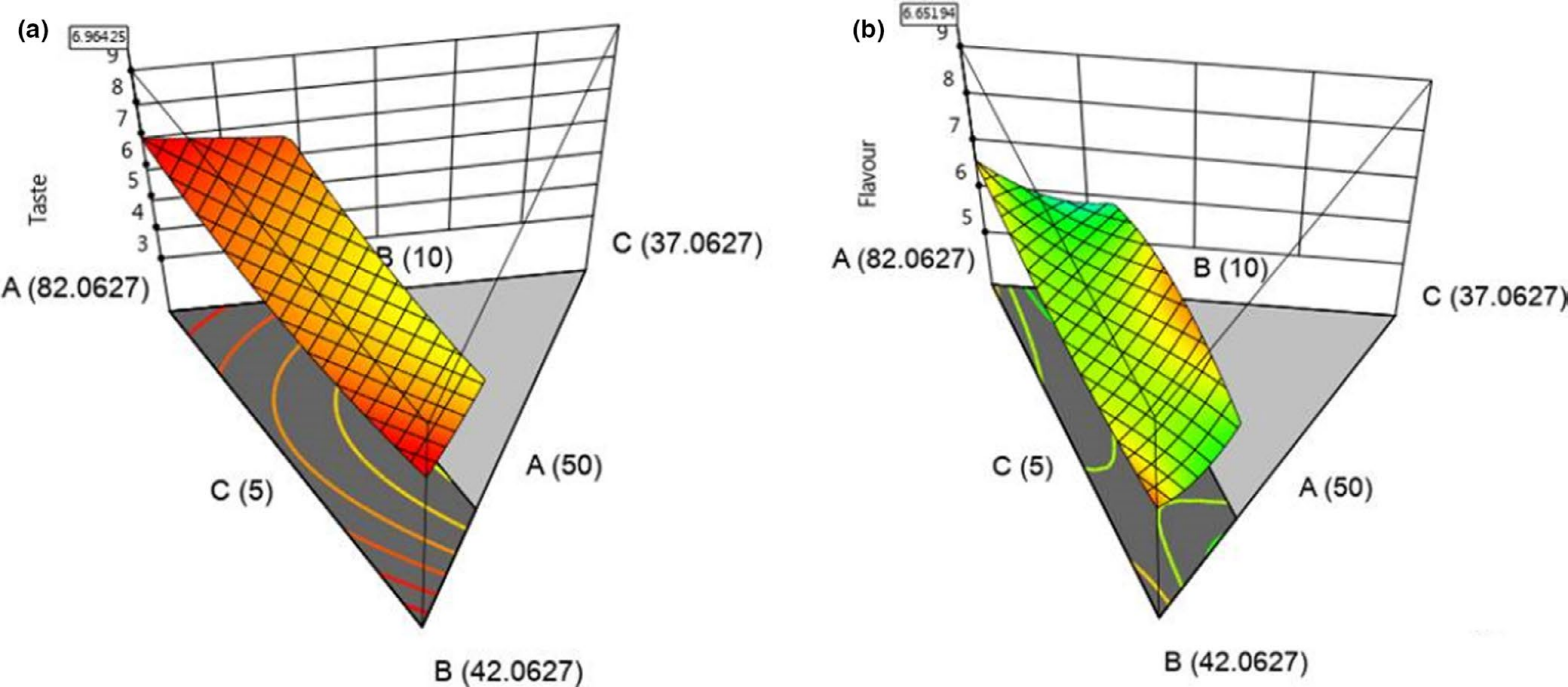
Response surface curves for the effect of ingredient proportions on (a) taste and (b) flavor changes in bean‐based composite extruded snacks and their optimal values. A, beans; B, maize; and C, OFSP; where amaranth was kept constant at 2.94 g/100 g of total ingredients

Sensory characteristics are key determinants of the acceptability of food among consumers (Carvalho et al.., 2013), and therefore can be used to determine the degree of liking or dislike for a new product. Based on hedonic rating of 1–9, mean scores for sensory attributes >5 (Table 3; Figure 2) were obtained indicating that appearance, taste, flavor, and overall acceptability attributes were moderately liked by panelists. Moderate low texture scores were recorded, and these could be attributed to the protein‐starch interactions (Yu, Liu, Tang, Shen, & Liu, 2017) or the starch‐fiber interactions (Robin, Théoduloz, & Srichuwong, 2015) which tend to limit expansion of extrudates. These findings agree with Allen, Carpenter, and Walsh (2007), who observed reduction in expansion of extrudates due to increases in protein in the raw material.

**TABLE 3:** Sensory scores for bean‐based extruded composite snack formulations

Exp. run	Ingredient proportions				Sensory scores				
	Beans (%)	Maize (%)	OFSP (%)	Amar (%)	Hardness	Crunchiness	Taste	Flavor	OA
1	50.00	34.33	10.66	5.01	7.12 ± 0.21^a^	7.25 ± 0.17^a^	6.75 ± 0.24^ac^	6.90 ± 0.21^a^	7.06 ± 0.20^abcde^
2	50.00	34.33	10.66	5.01	6.46 ± 0.24^a^	6.96 ± 0.22^a^	6.17 ± 0.23^abcd^	6.00 ± 0.23^abcd^	6.56 ± 0.20^abcde^
3	50.00	34.33	10.66	5.01	6.52 ± 0.24^a^	6.37 ± 0.27^a^	6.58 ± 0.22^abcd^	6.50 ± 0.21^abcd^	6.65 ± 0.17^abcde^
4	50.00	45.00	5.00	0.00	6.96 ± 0.19^a^	7.19 ± 0.17^a^	6.08 ± 0.22^abcd^	6.04 ± 0.21^abcd^	6.31 ± 0.19^e^
5	52.14	22.86	15.00	10.00	6.98 ± 0.20^a^	7.12 ± 0.19^a^	6.62 ± 0.20^abcd^	6.56 ± 0.21^ac^	7.71 ± 0.92^a^
6	54.04	30.56	5.40	10.00	7.39 ± 0.14^a^	7.23 ± 0.15^a^	6.29 ± 0.22^abcd^	6.31 ± 0.23^abcd^	6.75 ± 0.16^abcde^
7	55.66	29.34	15.00	0.00	6.46 ± 0.24^a^	6.96 ± 0.22^a^	6.54 ± 0.21^abcd^	6.50 ± 0.21^abcd^	6.65 ± 0.17^abcde^
8	59.25	35.75	5.00	0.00	7.04 ± 0.19^a^	7.23 ± 0.19^a^	6.42 ± 0.23^abcd^	6.58 ± 0.22^ac^	6.79 ± 0.19^abcde^
9	60.66	14.34	15.00	10.00	6.60 ± 0.21^a^	6.60 ± 0.19^a^	6.10 ± 0.23^abcd^	6.04 ± 0.22^abcd^	6.54 ± 0.21^abcde^
10	60.94	24.06	5.00	10.00	6.90 ± 0.22^a^	6.88 ± 0.23^a^	6.33 ± 0.25^abcd^	6.45 ± 0.23^abcd^	6.71 ± 0.24^abcde^
11	65.55	17.70	15.00	1.75	6.65 ± 0.25^a^	7.08 ± 0.21^a^	6.33 ± 0.26^abcd^	6.35 ± 0.24^abcd^	6.17 ± 0.23^cde^
12	65.99	25.17	8.84	0.00	6.77 ± 0.19^a^	7.08 ± 0.21^a^	6.27 ± 0.21^abcd^	6.25 ± 0.21^abcd^	6.79 ± 0.20^abcde^
13	65.99	25.17	8.83	0.00	6.52 ± 0.22^a^	6.43 ± 0.24	6.42 ± 0.23^abcd^	6.58 ± 0.22^ac^	6.89 ± 0.23^abcde^
14	68.02	22.64	5.00	4.34	6.67 ± 0.21^a^	6.79 ± 0.21^a^	6.14 ± 0.25^abcd^	6.10 ± 0.24^abcd^	6.46 ± 0.18^abcde^
15	68.02	22.64	5.00	4.34	6.54 ± 0.24^a^	6.85 ± 0.25^a^	6.86 ± 0.20^a^	6.37 ± 0.19^abcd^	6.88 ± 0.19^abcde^
16	71.85	10.00	8.61	9.54	6.52 ± 0.24^a^	6.43 ± 0.24^a^	5.60 ± 0.28^cd^	5.67 ± 0.26^cd^	6.02 ± 0.23^bcde^
17	71.85	10.00	8.61	9.54	6.98 ± 0.19^a^	7.11 ± 0.20^a^	6.61 ± 0.20^abcd^	6.56 ± 0.21^ac^	6.54 ± 0.21^abcde^
18	72.35	10.00	13.95	3.70	6.46 ± 0.24^a^	6.71 ± 0.24^a^	6.77 ± 0.20^ac^	6.50 ± 0.21^abcd^	6.98 ± 0.18^abcde^
19	76.44	16.78	5.83	0.95	6.60 ± 0.22^a^	6.98 ± 0.17^a^	6.37 ± 0.20^abcd^	6.02 ± 0.21^abcd^	6.44 ± 0.15^abcde^
20	85.00	10.00	5.00	0.00	7.10 ± 0.20^a^	7.23 ± 0.20^a^	6.83 ± 0.21^a^	6.44 ± 0.19^abcd^	6.77 ± 0.20^abcde^

Note

Values are means and standard errors of means. *n* = 52 panelists.

Superscript letters in a column are significantly different (*p* < .05).

Abbreviations: Amar, amaranth; OA, overall acceptability.

Moderate to high flavor scores was observed in this study (Table 3). The flavor scores observed were perhaps due to food browning reactions during extrusion cooking which produce nice nutty smells desirable to consumers (Mohsen, Fadel, Bekhit, Edris, & Ahmed, 2009). Nyombaire et al. (2011) and Siddiq et al. (2013) on the other hand associated low flavor scores to the inherent beany flavors, not desired by consumers. Reports, however, show that the beany flavor from legumes can be removed by extrusion (Rocha‐Guzmán et al., 2006). In general, the moderate to high sensory scores from this study were indicative of acceptable bean‐based snack product to consumers.

As observed (Table 3) high beans content had less significant effect on the taste and flavor of extrudates (*R* ^2^ ≤ .7), implying that variation in these attributes was partly dependent on other factors. None of the formulations scored lower than 5, a score considered the lower limit of acceptability (Nyombaire et al., 2011), for any sensory attribute. Partial use of bean flour has previously been shown to give acceptable products (Anton et al., 2009; Nyombaire et al., 2011). In this study, 83% of the panelists rated the acceptability of formulations between 6 and 9, implying that the snacks were moderately to highly acceptable.

#### 3.4.2 Acceptability of developed snack formulation among school children

The taste and texture average scores (Table 4) showed that a formulation with moderate maize content (25%) and relatively high beans content (66%) was liked most by children. It is likely that high proportion of maize in the formulation resulted into more acceptable snacks. The taste and textural characteristics of the extruded snack were likely due to interaction between protein and starch in the ingredients. Yu et al. (2017) explained that protein‐starch interactions modify the expanded gel outer characteristics, possibly improving its texture. Chaiyakul, Jangchud, Jangchud, Wuttijumnong, and Winger (2008) noted that even though protein increases the hardness, it improves the crispiness and noise intensity of extruded snacks. The increase in hardness might be due to reduced extrudate expansion (Hagenimana, Ding, & Fang, 2006; Yu et al., 2017).

**TABLE 4:** Bean‐based composite extruded snack average acceptability scores provided by school children

Ingredient proportions				Children's average sensory scores	
Bean	Maize	OFSP	Amaranth	Taste	Texture (hardness)
65.99	25.17	8.83	0.00	4.25 ± 1.18^a^	3.91 ± 1.09^a^
85	10.00	5.00	0.00	3.98 ± 1.20^b^	3.85 ± 1.07^a^

Note

Means in each column with different superscripts are significantly different (*p* < .05). Values are means ± standard errors of means. *n* = 112 children.

Superscript letters in a column are significantly different (*p* < .05).

#### 3.4.3 Optimal bean‐based composite snack formulation and product response values

Numeric optimization based on protein, dietary fiber, iron, zinc content; taste, protein digestibility, instrumental hardness, and crispiness (Area‐FD) of extrudate yielded an optimal formulation with global desirability index of 0.97. A desirability index of 0.97 for a snack formulation containing 82% beans in total ingredients and providing 20.4 g protein and 4.8 mg iron for every 100 g of snack with 5.7 average taste score possibly suggests adequacy in nutritional value and acceptability (Table 5).

**TABLE 5:** Attribute desirable values and optimal desirability indices

Run	Ingredient proportions (%)				Protein (g)	Nutrient composition & digestibility per 100 g				Texture properties			D
	Bean	Maize	OFSP	Amar.		DF (g)	Iron (mg)	Zinc (mg)	Taste	Flav	Hd (*N*)	A‐FD (*N*.mm)	
1	82.1	10.0	5.0	2.9	22.7	13.8	6.1	3.4	7.0	6.7	26.6	118.8	0.97
2	84.8	10.2	5.0	0.0	23.0	14.0	6.1	3.4	6.5	6.8	46.8	180.3	0.81
3	73.5	10.0	15.0	1.5	20.4	12.7	5.4	3.0	6.7	5.4	41.2	161.4	0.66
4	67.5	27.5	5.0	0.0	19.8	12.9	5.3	3.1	6.0	6.4	37.2	168.3	0.65

Note

A‐FD, area‐force dimension ratio; D, global desirability index; DF, dietary fiber; Flav, flavor; Hd, hardness.

Actual values (Table 6) for composition, sensory, and instrumental texture were relatively low compared with and significantly different from the predicted values (*p* < .05), which could be attributed to error and nutrient losses during the preprocessing of ingredients. In addition, it is likely that the nutrient composition values in databases used were relatively high compared with the raw material composition. The taste and flavor scores of the optimal bean‐based snack (Table 6) were low compared with scores for commercial extruded corn snack (7.18 and 7.38, respectively). This could be suggestive of need for improvement of product palatability attributes. Panelists especially recommended improvement of color and attractiveness of the extruded bean‐based snack.

**TABLE 6:** Predicted and actual optimal values of the bean‐based snack nutrient composition, sensory, and textural properties

Snack attribute	Goal	Predicted values^a^	Actual values^b^	*p*‐values
Protein (g/100 g)	Maximize	22.66	20.38	.03
Dietary fiber (g/100 g)	Maximize	13.78	4.12	<.01
Iron (mg/100 g)	Maximize	6.07	4.83	<.01
Zinc (mg/100 g)	Maximize	3.37	1.51	<.01
Taste	Maximize	6.96	5.65	<.01
Flavor	Is in range	6.65	5.78	<.01
Hardness (*N*)	Minimize	26.63	13.53	<.01
Area‐FD (*N*.mm)	Minimize	118.78	75.66	<.01

^a^ Predicted optimal values as generated by numeric optimization using Design Expert mixture design.

^b^ Actual optimal values as generated by experimental analysis

### 3.5 Nutrient density and nutrient contribution from the extruded bean‐based snack

Nutrients derived from consuming the optimal bean‐based snack were computed from the nutrient density per 100 kcal and direct comparison with the recommended daily intake (RDI) for children aged 4–12 years (Swanepoel et al., 2018). Estimation of the nutrient density and nutrient contribution showed that from 100 g snack serving of the optimal extruded bean snack if consumed would provide at least 60%, 48%, or 19% of the RDI for protein, iron, and zinc, respectively, for 9‐ to 12‐year‐old and more than 100% for 4–8 year children (Table 7). A typical 40 g serving would provide 42.9%, 19.3%, and 12.1% of protein, iron, and zinc of RDI, respectively, to children aged 4–8 years. The nutrient density of the optimal snack formulation in g for macronutrients (and μg for minerals) per 100 kcal of energy varied between 0.41 and 5.51 depending on nutrient. Therefore, the optimal formulation would effectively contribute to reducing nutrient deficiencies among school‐age children.

**TABLE 7:** Nutrient contribution derived from consuming the optimal snack formulation

Age (years)	Recommended daily nutrient intake				Percentage daily nutrient contribution by					
					40 g snack serving			100 g snack serving		
	Protein (g/day)	Energy (kcal/day)	Iron, (mg/day)	Zinc, (mg/day)	Protein	Iron	Zinc	Protein	Iron	Zinc
4–8	19	1,742	10	5	42.91	19.32	12.08	107.30	48.30	30.20
9–13	34	2,279	8	8	23.98	24.15	7.55	59.94	60.38	18.88

## 4 CONCLUSION

A bean‐based extruded snack containing 82% beans, 10% maize, 5% OFSP, and 3% amaranth was found to exhibit the most desirable nutritional and acceptability properties. The optimal formulation was found to contain 20.4 g of protein, 48.3 mg of iron, and 15.1 mg zinc per 100 g. The study demonstrated that extrusion cooking can be used to develop acceptable and nutritious composite snacks from iron‐rich beans. Bean flours considerably increased the total protein (2.5‐fold), iron, and zinc content in the snack when compared to extruded plain corn snacks. Thus, ingredient complementation can strategically be applied in the development of extruded bean‐based food products to boost nutritional quality and sensory acceptability.

## CONFLICT OF INTEREST

The authors declare no conflict of interest.
